# Supplementary material for: Loss of Mitochondrial Tusc2/Fus1 Triggers a Brain Pro-Inflammatory Microenvironment and Early Spatial Memory Impairment
Source: Int J Mol Sci. 2024 Jul 5;25(13):7406. doi: 10.3390/ijms25137406 (PMC11242373; doi:10.3390/ijms25137406)
Supplement: Supplementary file 1 [file ijms-25-07406-s001.zip › ijms-3018134-supplementary.pptx]

## Slide 1
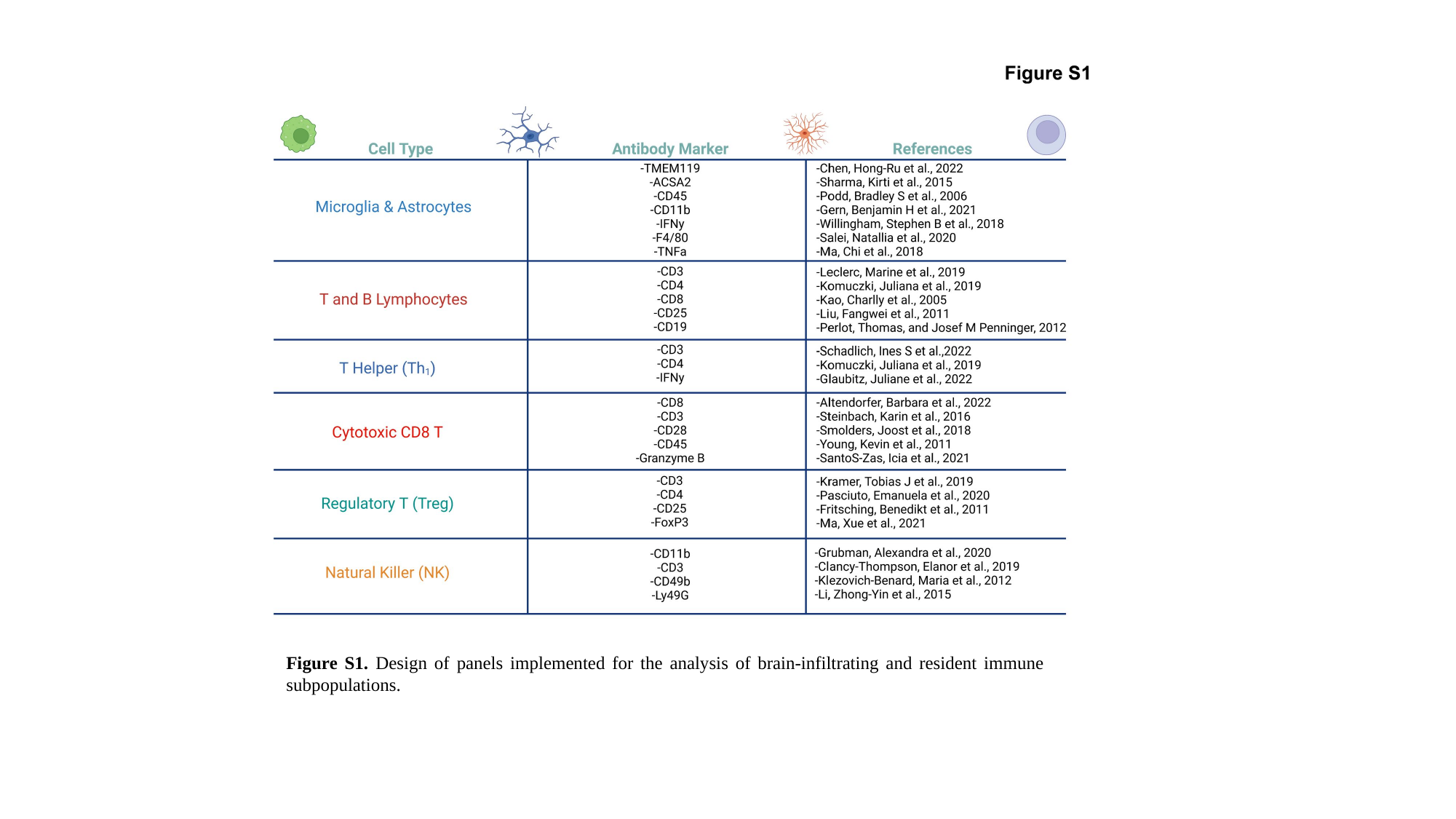

Figure S1. Design of panels implemented for the analysis of brain-infiltrating and resident immune subpopulations.

## Slide 2
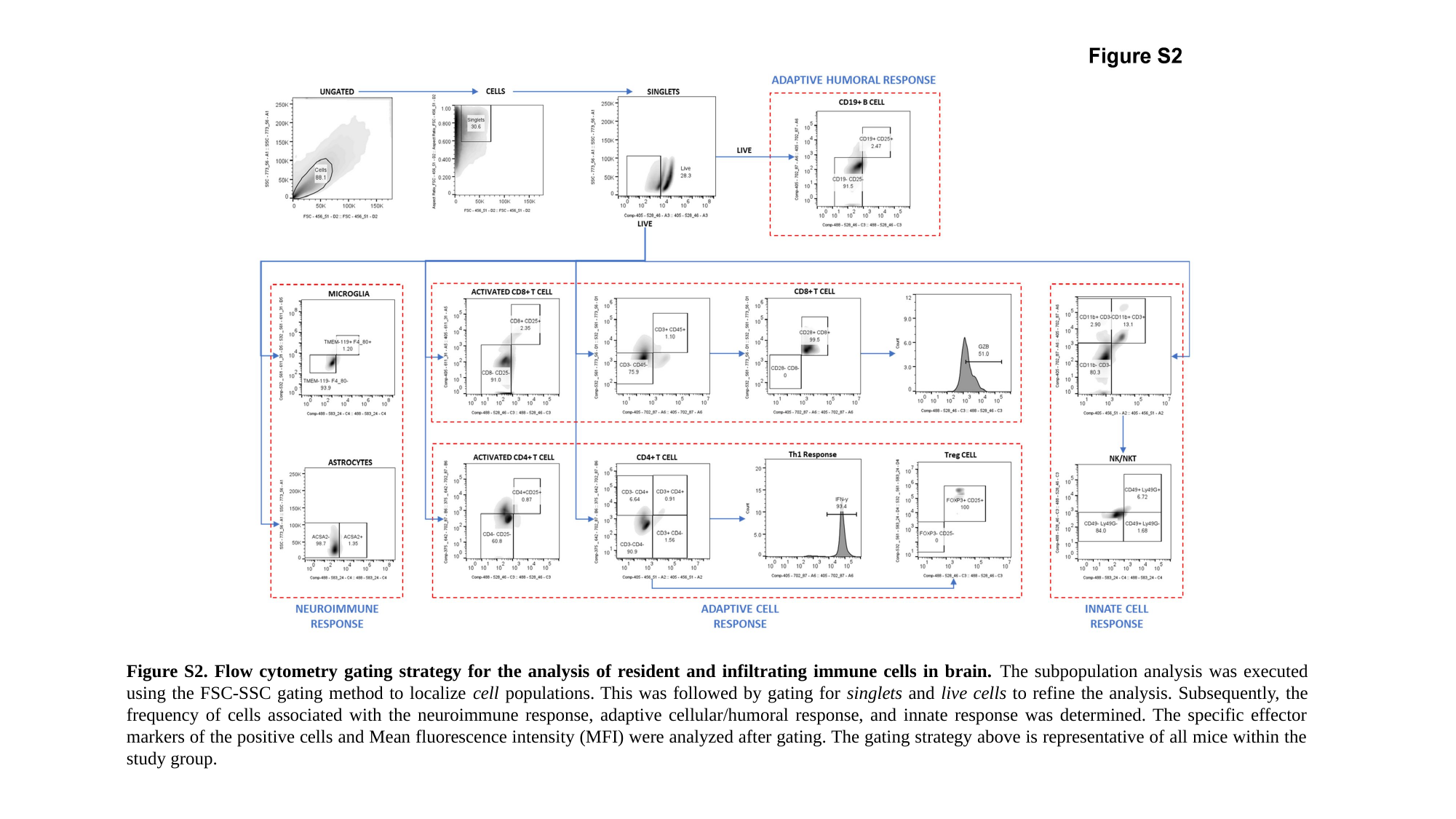

Figure S2. Flow cytometry gating strategy for the analysis of resident and infiltrating immune cells in brain. The subpopulation analysis was executed using the FSC-SSC gating method to localize cell populations. This was followed by gating for singlets and live cells to refine the analysis. Subsequently, the frequency of cells associated with the neuroimmune response, adaptive cellular/humoral response, and innate response was determined. The specific effector markers of the positive cells and Mean fluorescence intensity (MFI) were analyzed after gating. The gating strategy above is representative of all mice within the study group.
